# Delays in Pediatric Studies Required Under the US Pediatric Research Equity Act

**DOI:** 10.1001/jamanetworkopen.2025.41176

**Published:** 2025-10-30

**Authors:** Rylee McGonigle, Florence T. Bourgeois

**Affiliations:** 1 Harvard-MIT Center for Regulatory Science, Harvard Medical School, Boston, Massachusetts; 2 Pediatric Therapeutics and Regulatory Science Initiative, Computational Health Informatics Program, Boston Children's Hospital, Boston, Massachusetts; 3 Department of Pediatrics, Harvard Medical School, Boston, Massachusetts

## Abstract

This cohort study investigates delays in Pediatric Research Equity Act–required studies and issuance of noncompliance letters by the Food and Drug Administration.

## Introduction

The Pediatric Research Equity Act (PREA) was implemented in 2003 to expand prescribing information for pediatric patients by allowing the US Food and Drug Administration (FDA) to require that sponsors perform pediatric studies for certain new drugs.^1^ Prior research assessing this policy found that only approximately one-third of PREA studies were completed after a median of 7 years, resulting in prolonged periods of potential off-label use in pediatric populations.^2^ While the FDA monitors PREA study progress, it can issue noncompliance letters only after sponsors fail to meet final study submission deadlines. We performed a longitudinal analysis evaluating PREA study delays and issuance of noncompliance letters.

## Methods

In accordance with the Common Rule, this cohort study was exempt from institutional review board review given that human participants were not involved. The study followed the STROBE reporting guideline. We reviewed all novel drugs approved by the FDA Center for Drug Evaluation and Research from 2015 to 2019 and identified pediatric postmarket studies required under the PREA using approval letters in the Drugs@FDA database. The study period allowed for a minimum of 5 years of follow-up through December 31, 2024. We collected study characteristics from study descriptions in approval letters (eMethods in Supplement 1). To determine whether a study was delayed during trial conduct, we examined study statuses using the FDA Postmarketing Requirements and Commitments Database.^3^ For all studies that had been delayed, we determined whether a PREA noncompliance letter was issued.^4^

We used χ^2^ tests and *t* tests to compare studies with and without delays. Analyses were conducted in R Studio version 2024.12.1 + 563 (RStudio). A 2-sided *P* value < .05 was considered statistically significant.

## Results

Among 220 novel drugs approved by the FDA between 2015 and 2019, 62 drugs (28.2%) had a total of 137 pediatric studies required under the PREA at the time of approval. Most studies were primary efficacy (64 studies [46.0%]) or safety (55 studies [40.1%]) studies, and they were concentrated across a few therapeutic areas, with approximately one-half studying nervous system agents (39 studies [28.5%]) or antiinfectives (30 studies [21.9%]) (Table).

**Table.** Pediatric Research Equity Act–Required Study Characteristics

Characteristic	Total studies, No. (column %) (N = 137)	Studies by delay status, No. (row %)		*P* value
		Ever delayed (n = 65)	Never delayed (n = 72)	
Drug type				
Small molecule drug	105 (76.6)	53 (50.5)	52 (49.5)	.28
Biologic	32 (23.4)	12 (37.5)	20 (62.5)	
Study type				
Primary efficacy study	63 (46.0)	32 (50.8)	31 (49.2)	.74
Primary safety study	55 (40.1)	24 (43.6)	31 (56.4)	
Primary PK/PD study	19 (13.9)	9 (47.4)	10 (52.6)	
Youngest pediatric age group				
Neonate (0 to <1 mo)	18 (13.1)	9 (50.0)	9 (50.0)	.43
Infant (1 mo to <2 y)	26 (19.0)	11 (42.3)	15 (57.7)	
Early childhood (2 to <6 y)	21 (15.3)	10 (47.6)	11 (52.4)	
Late childhood (6 to <12 y)	48 (35.0)	26 (54.2)	22 (45.8)	
Adolescent (12 to <18 y)	20 (14.6)	9 (45.0)	11 (55.0)	
Unspecified	4 (2.9)	0 (0.0)	4 (100.0)	
Therapeutic area^a^				
Nervous system	39 (28.5)	13 (33.3)	26 (66.7)	.003
Anti-infectives for systemic use	30 (21.9)	12 (40.0)	18 (60.0)	
Alimentary tract and metabolism	25 (18.2)	21 (84.0)	4 (16.0)	
Immunomodulating agents	13 (9.5)	7 (53.9)	6 (46.1)	
Hematologic agents	11 (8.0)	5 (45.5)	6 (54.5)	
Other^b^	19 (13.9)	7 (36.8)	12 (63.2)	

Abbreviation: PK/PD, pharmacokinetic/pharmacodynamic.

^a^ Based on the Anatomical Therapeutic Chemical classification system.

^b^ Other includes all therapeutic groups constituting less than 3% of the total study count: dermatologicals (4 studies [2.9%]), cardiovascular (4 studies [2.9%]), various (4 studies [2.9%]), respiratory system (3 studies [2.2%]), systemic hormonal preparations (3 studies [2.2%]), and antiparasitic products, insecticides, and repellents (1 study [0.7%]).

There were 65 studies (47.4%) pertaining to 34 drugs (54.8%) that experienced delays. Delays did not differ significantly by drug type, study type, or age group but varied by therapeutic area, ranging from 13 studies (33.3%) of nervous system agents to 21 studies (84.0%) of alimentary tract drugs (*P* = .003). Studies with delays took a mean of 2.2 years (95% CI, 1.12-3.20 years; *P* < .001) longer to complete than those without delays.

The FDA issued noncompliance letters for 9 studies that failed to meet final submission deadlines (13.8% of studies with delays). Of these, 2 studies were subsequently completed while 7 studies remained delayed after a median (range) of 4.1 (0.68-4.76) years after the letter.

## Discussion

In this retrospective cohort study of pediatric studies required under the PREA, delays in study completion were common, with nearly half delayed during study conduct. Noncompliance letters were provided for a few delayed studies after they missed reporting deadlines, although most remained delayed years after the letter was issued. A study limitation is the lack of publicly available information on factors contributing to delays and on potential interactions between the FDA and sponsors beyond letters.

Enforcement activities may be more effective if available to the FDA when initial study milestones are missed. In addition, while the FDA has the authority to impose financial penalties for sponsors not complying with adult postmarket study requirements, it is legally prohibited from doing so for pediatric studies.^5^ A bill was introduced in the US Congress in 2023 to remove this exemption and align enforcement tools across pediatric and adult policies.^6^ Although the bill was not enacted, our findings underscore the critical need for this and other legislative reforms to increase timely and rigorous conduct of pediatric drug trials.

## Notes

Supplement 1.
eMethods.

eReferences.

Supplement 2.
Data Sharing Statement
