# Supplementary material for: Delays in Pediatric Studies Required Under the US Pediatric Research Equity Act
Source: JAMA Netw Open. 2025 Oct 30;8(10):e2541176. doi: 10.1001/jamanetworkopen.2025.41176 (PMC12576482; doi:10.1001/jamanetworkopen.2025.41176)
Supplement: Supplement 1. — eMethods. eReferences. [file jamanetwopen-e2541176-s001.pdf]

Supplemental Online Content

McGonigle R, Bourgeois FT. Delays in pediatric studies required under the US Pediatric Research Equity Act. *JAMA Netw Open*. 2025;8(10):e2541176. doi:10.1001/jamanetworkopen.2025.41176

eMethods.

eReferences.

This supplemental material has been provided by the authors to give readers additional information about their work.

© 2025 McGonigle R et al. *JAMA Network Open*.

eMethods

Data Collection on PREA studies

Information on PREA studies were obtained from FDA drug approval letters, which include a specific section detailing pediatric studies required under PREA. Data were collected on study type (primary efficacy, safety, or pharmacokinetic/pharmacodynamic study), pediatric age groups to be studied (neonates [0 - <1 month], infants [1 month - <2 years], early childhood [2 - <6 years], late childhood [6 - <12 years], and adolescent [12 - <18 years]), and the drugs' therapeutic area, based on the Anatomical Therapeutic Chemical classification system.[1] Time to study completion was calculated using the date of study issuance and date of study submission reported in the FDA Postmarketing Requirements and Commitments Database,[2] stratified on whether the study had ever been delayed.

Determination of PREA Study Status

We used the FDA Postmarketing Requirements and Commitments Database to collect information on the status of studies through December 31, 2024.[2] Study statuses are assigned by the FDA and categories include pending, ongoing, delayed, submitted, fulfilled, and released. Since studies can change status over time, we reviewed archived quarterly versions of the database (available on the FDA website) to determine whether a study had been delayed at any time since study request.

© 2025 McGonigle R et al. *JAMA Network Open*.

eReferences

1. World Health Organization. Anatomical Therapeutic Chemical (ATC) Classification. World Health Organization. Accessed March 21, 2025. https://www.who.int/tools/atc-ddd-toolkit/atc-classification

2. US Food and Drug Administration. Postmarketing Requirements and Commitments: Searchable Database. US Food and Drug Administration. Accessed March 21, 2025. https://www.accessdata.fda.gov/scripts/cder/pmc/index.cfm

© 2025 McGonigle R et al. *JAMA Network Open*.
