# Supplementary material for: Delays in Pediatric Studies Required Under the US Pediatric Research Equity Act
Source: JAMA Netw Open. 2025 Oct 30;8(10):e2541176. doi: 10.1001/jamanetworkopen.2025.41176 (PMC12576482; doi:10.1001/jamanetworkopen.2025.41176)
Supplement: Supplement 2. — Data Sharing Statement [file jamanetwopen-e2541176-s002.pdf]

Data Sharing Statement

McGonigle. Delays in Pediatric Studies Required Under the US Pediatric Research Equity Act. JAMA Netw Open. Published online October 30, 2025. doi:10.1001/jamanetworkopen.2025.41176

Data
Data available: Yes
Data types: Data (not involving human participants)
How to access data: All data are publicly available through online FDA resources.
When available: With publication

Supporting Documents
Document types: None

Additional Information
Who can access the data: Any investigator
Types of analyses: Any analyses
Mechanisms of data availability: All data are publicly available through online FDA resources.
